# Alexithymia and estimated 10-year cardiovascular disease risk in healthy adults: a community-based cross-sectional study

**DOI:** 10.3389/fpsyg.2024.1504143

**Published:** 2024-12-04

**Authors:** Francesco Vadini, Roberta Lanzara, Ornella Iuliani, Gianna Pia Affaitati, Piero Porcelli

**Affiliations:** ^1^Department of Psychology, University “G. d’Annunzio” of Chieti-Pescara, Chieti, Italy; ^2^Department of Oncology and Hematology, Pescara General Hospital “Santo Spirito”, Pescara, Italy; ^3^Department of Innovative Technologies in Medicine and Dentistry, University “G. d’Annunzio” of Chieti-Pescara, Chieti, Italy

**Keywords:** alexithymia, blood donors, cardiovascular risk, quality of life, psychosomatic

## Abstract

**Background:**

This cohort study aimed to explore whether and to what extent alexithymia would be associated with cardiovascular disease (CVD) risk over an estimated 10-year period, over and above established clinical cofactors (i.e., depressive symptoms, quality of life, sociodemographic, anthropometric, lifestyle, and biological data), in a low-risk population of blood donors.

**Methods:**

A sample of 1,021 adult Italian blood donors (age 46.9 ± 8.39; 61.2% men) was consecutively recruited. The 10-year-CVD risk score was estimated using the CUORE risk score (CRS). Sociodemographic, lifestyle, anthropometric, biological, and psychological (i.e., quality of life, depressive symptoms, and alexithymia) CVD risk data were assessed using validated self-report measures or clinical records.

**Results:**

As expected, most participants (78.5%) had a low CVD risk (CRS < 3%) and an overall low-risk profile for all the parameters. Compared with subjects at low risk of CVD (*n* = 911, 78.5%), those with high risk (i.e., rated ≥3 on CUORE risk assessment; *n* = 250, 21.5%) reported higher levels of alexithymia (*p* < 0.001). Subjects with higher alexithymia (*n* = 236, 23.1%) reported higher levels of psychosocial impairment, depressive symptoms, and biological risk variables for CVD. Alexithymia was significantly associated with 10-year CVD risk (OR = 1.02, 95% CI = 1.01–1.04, *p* = 0.009), even after adjusting for key sociodemographic and clinical risk variables.

**Conclusion:**

Although limited by the cross-sectional design, this study is the first to show that alexithymia leads to a higher risk for 10-year CVD estimate in healthy subjects with low-risk profile, regardless of known biomarkers and traditional CVD risk factors.

## 1 Introduction

Cardiovascular disease (CVD) is the leading cause of premature mortality worldwide, accounting for more than 17 million estimated deaths per year (Lindstrom et al., 2022). To date, biomedical risk factors such as obesity (Franks et al., 2010), cholesterol (Burger et al., 2024), C-reactive protein (Prasad, 2024), blood-pressure level (Ettehad et al., 2016), and metabolic syndrome (MetS) (Kurniawan, 2024), as well as lifestyle (e.g., physical inactivity, smoking, alcohol consumption, unhealthy diet) and psychological variables (Bogle et al., 2016; Carola et al., 2024; Emdin et al., 2016; Rozanski, 2014) have been studied extensively. Among psychological variables, specifically depression has been largely investigated and associated with poor quality of life, higher healthcare costs, and increased risk of new cardiac events and mortality (Krittanawong et al., 2023), even when controlling for known risk factors (unhealthy lifestyle, body mass index [BMI], hypertension, diabetes, and socioeconomic status) (Gan et al., 2014; Meijer et al., 2011). It is notable that also minor depression (Freedland et al., 2016) and emotional distress—though at a subthreshold diagnostic level—may increase the risk of CVD morbidity and mortality (Lissåker et al., 2019; Norlund et al., 2018).

Alexithymia is one of the psychological factors investigated in CVD (e.g., Montisci et al., 2023; Sancassiani et al., 2023) and other medical conditions (Luminet et al., 2018). Alexithymia is a personality dimension defined as a deficit in the cognitive processing of emotions. It is defined by two higher-order factors, namely a deficit of affect awareness (difficulty in identifying and describing feelings) and operatory thinking (externally oriented thinking and poor imaginal process) (Taylor and Bagby, 2004). Current evidence shows that alexithymia may affect health in a number of ways, as it is associated with affective states (e.g., depressive symptoms), maladaptive behaviors (e.g., altered eating behavior), emotional dysregulation related psychopathology (through somatosensory amplification), post traumatic shutdown of emotions (e.g., acute reactions to illness), and altered autonomic, endocrine and immune activity leading to tissue damage (e.g., vulnerability to inflammatory processes) (for an extensive review, see Luminet et al., 2018). Alexithymia has been associated with known risk factors for cardiovascular disease such as biochemical and anthropometric alterations (e.g., high levels of cholesterol, triglyceride, glucose, BMI, resting sympathetic activity, heart rate, blood pressure, and blood pressure reactivity) and unhealthy behaviors (e.g., sedentary lifestyles, and poorer nutrition and glycemic control) (Aluja et al., 2020; Sancassiani et al., 2023). Furthermore, alexithymia was also found to be an independent factor increasing the risk of CVD death (Carta et al., 2022; Tolmunen et al., 2010; Vadini et al., 2019). More specifically, Carta et al. (2022) investigated the relationship between 10-year mortality and alexithymia among people with myocardial infarction. Among people with high alexithymia in 2011, a higher risk of early death in 2021 was found (RR = 5.75). In a prospective study, Vadini et al. (2019) found that alexithymia was an independent predictor of carotid plaques (OR = 4.93) at baseline and cardiovascular events (HR = 3.66) and mortality (HR = 3.93) at follow-up in patients with HIV. Tolmunen et al. (2010) have pointed out that subjects with high alexithymia have both higher BMI and systolic blood pressure, are more frequently smokers, perform less physical activity, and report a 2.3% increase in CVD mortality risk for each 1-point increase on the Toronto Alexithymia Scale. Other studies report similar results, thus providing further evidence of the role of alexithymia in triggering several CVD risk factors linked both to unhealthy lifestyle and to the autonomic alterations due to emotional dysregulation (Carta et al., 2013; Marchesi et al., 2015; Meloni et al., 2016; Montisci et al., 2023; Sancassiani et al., 2023). However, the role of alexithymia in CVD is still debated. For example, it remains unclear whether there is a significant association between behavioral and physiological CVD risk factors in healthy populations (Luminet et al., 2018; Montisci et al., 2023).

The present study aimed to explore whether alexithymia would be associated with increased CVD risk over an estimated 10-year period in a low-risk population of blood donors. Specifically, the aim of the study was twofold: (a) to investigate whether sociodemographic and anthropometric data, lifestyle, biological markers, and psychological characteristics would differ between subjects with high and low cardiovascular risk and between subjects with high and low levels of alexithymia; (b) to explore whether and to what extent alexithymia would be associated with increased CVD risk, over and above established clinical cofactors (i.e., depressive symptoms and quality of life, sociodemographic, anthropometric, lifestyle, and biological data). Blood donors are screened for good physical health and undergo blood donation strictly voluntarily, thus showing an active prosocial attitude. Although this was the first study investigating the association between alexithymia and CVD risk in a blood donor population, we expected that: (a) subjects with higher CVD risk would report higher alexithymia and subjects with higher levels of alexithymia would report higher CVD risk; (b) alexithymia would be associated with increased CVD risk over and above other relevant co-factors.

## 2 Materials and methods

### 2.1 Population and study design

In this cross-sectional study, a sample of *N* = 1,610 volunteer blood donors were consecutively enrolled at the Blood Transfusion Unit of the General Hospital of Pescara (Italy) between June 2015 and July 2017. All the participants were medically tested for major severe diseases and major contraindications for donating blood. The study protocol was administered during the medical examination to assess eligibility for blood donation. To maximize ecological validity, we used the standard inclusion criteria for blood donation: age range 35–69 with good physical health and a proactive social attitude. Subjects were excluded if they had certified medical or psychiatric morbidity (such as mental major impairment, drug or alcohol abuse), impairment in cognitive functions, were not fluent Italian speakers, or were pregnant.

**Figure 1:**
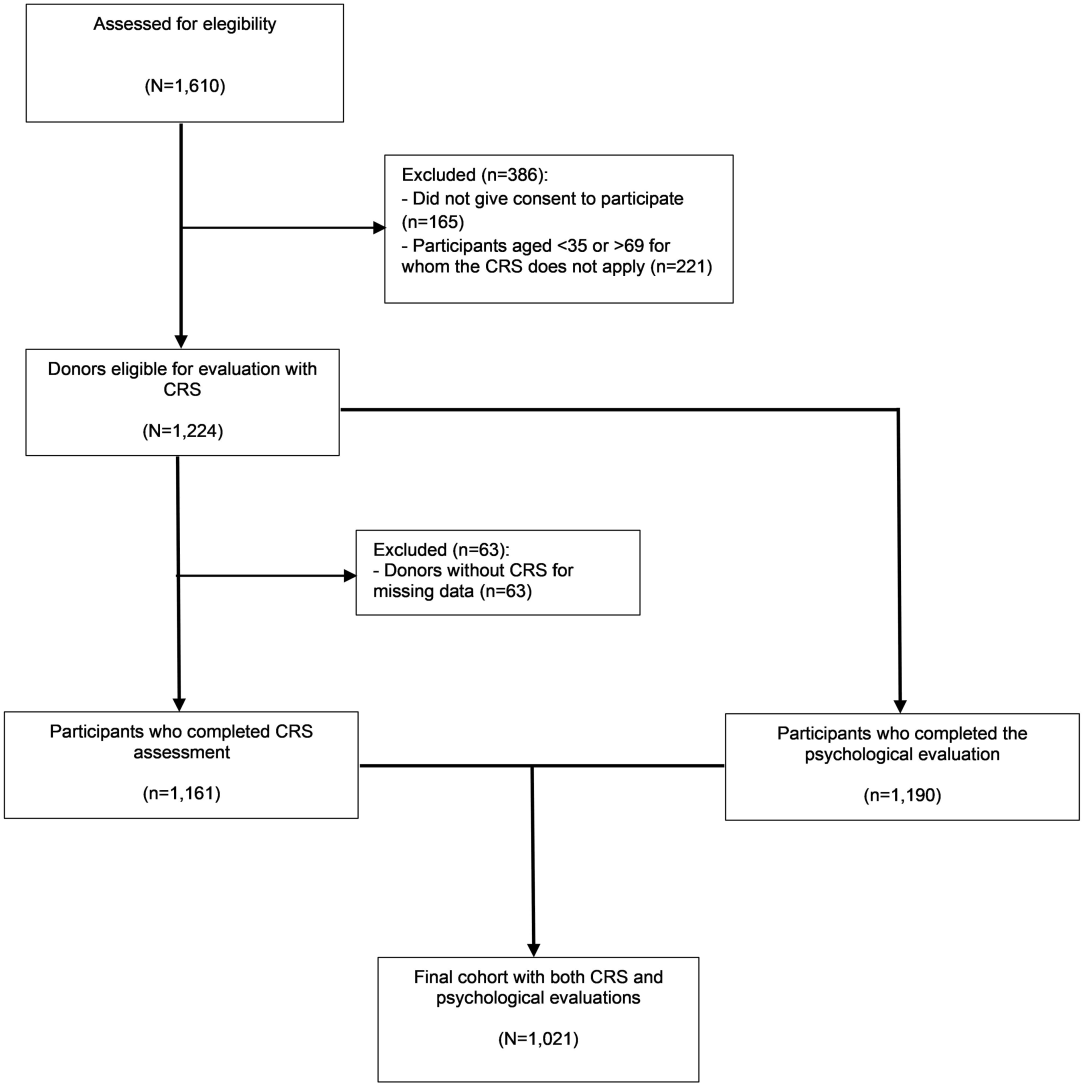
Flow chart describing the participation in the study (created using MS Office).

Figure 1 shows the flowchart of participation in the study. Of the 1,610 blood donors assessed for eligibility, 165 (10.2%) refused to participate for lack of time and 221 (13.7%) were of non-eligible age for evaluation with CRS, so they were excluded. Data were collected from 1,161 participants for the CRS assessment, from 1,190 participants for the psychological assessment, and from 1,021 participants for both assessments. Only the latter subjects entered the multivariate analysis. According to the sociodemographic variables, no significant difference was found between included and excluded participants.

The study protocol was ethically approved by the local institutional review board of the Oncology Hematology Department of Spirito Santo Hospital in Pescara. Written informed consent was obtained from the participants in accordance with the Declaration of Helsinki.

### 2.2 Measures

#### 2.2.1 Socio-demographic data

Socio-demographic data (i.e., age, gender, years of education, occupation, marital status) were collected using an *ad-hoc* questionnaire.

The socioeconomic status (SES) was classified as low (manual workers and skilled/unskilled workers including farmers with primary education level [5 years]), middle (non-manual employees and clerks with secondary education level [8–12 years]), or high (professionals, executives, administrators, and entrepreneurs with tertiary level of education) status. Low education was defined as ≤13 years of school attendance (corresponding to compulsory education in Italy and to the <25th centile within the study sample) and high education as >13 years of school attendance.

#### 2.2.2 Psychological factors

##### 2.2.2.1 Alexithymia

Alexithymia was assessed using the 20-item Toronto Alexithymia Scale (TAS-20) which is rated on a 5-point Likert scale ranging from 1 (strongly disagree) to 5 (strongly agree). In addition to the total, the TAS-20 yields score for difficulty in identifying feelings (DIF), difficulty in describing feelings (DDF), and externally oriented thinking (EOT). The scale is considered the standard measure for alexithymia given the internal reliability (Cronbach’s *α* coefficients of 0.80 to 0.83 for the total score) of its psychometric properties, the construct validity, and the factor structure (Bagby et al., 2020). Subjects scoring >60 are defined as highly alexithymic (Bressi et al., 1996; Taylor et al., 1999). The cut-off score of >60 used in clinical studies was found to be a restrictive threshold for our healthy population, with very few blood donors (7.5%) classified as alexithymic. Therefore, we divided the study population into two groups, high (HA) and low alexithymia (LA) groups, using the least restrictive cut-off corresponding to TAS-20 score > 49, a threshold previously employed in large non-clinical population studies (Grabe et al., 2010). Interestingly, this threshold in our cohort coincides with the 75th percentile of TAS-20 score. Furthermore, the TAS-20 > 49 threshold was used as a threshold particularly predictive of subclinical cardiometabolic risk factors in large population-based studies (Grabe et al., 2010; Vadini et al., 2019). Within the sample used in this analysis, Cronbach’s *α* was 0.83 for the total scale, 0.85 for the DIF, 0.70 for the DDF, and 0.56 for the EOT subscales.

##### 2.2.2.2 Depressive symptoms

Depressive symptoms were assessed using the 21-item Beck Depression Inventory-II (BDI-II) (Beck et al., 1996), in which items are rated on a 4-point scale ranging from 0 to 3 and the total score ranges from 0 to 63. Higher scores correspond to greater severity. Prior research has indicated that this measure is internally consistent and reliable (Cronbach’s α coefficient of 0.89), has discriminant power, and correlates highly with other measures of depressive symptoms and depression-related constructs (for a review, see Whisman and Richardson, 2015). According to the guidelines, we used a score > 13 as a threshold for detecting depressive symptoms (Ghisi et al., 2006). Within the sample used in this analysis, Cronbach’s *α* was 0.86.

##### 2.2.2.3 Health-related quality of life

Psychosocial functioning was assessed using the Short Form-12 Health Survey (SF-12) scale (Gandek et al., 1998). The SF-12 is a shortened version of the 36-item questionnaire (SF-36), proven to be more practical in large samples when an overall estimate of physical and mental health is needed (Apolone and Mosconi, 1998; Melville et al., 2003). The scale consists of 12 questions about both physical (PCS, physical component summary) and mental functioning (MCS, mental component summary). Higher scores indicate a better psychosocial functioning. The SF-12 has been used in various settings and study populations, thus it is considered a valid and reliable tool with a good internal reliability (Cronbach’s α coefficients of 0.72 to 0.89) (Andriopoulos et al., 2013; Rawal et al., 2013). Within the sample used in this analysis, Cronbach’s α was 0.83 for MCS and 0.88 for PCS.

#### 2.2.3 Anthropometric, health and lifestyle behavior data

##### 2.2.3.1 Blood pressure

Resting blood pressure (BP) was measured after 5 min in a seated position with an automated BP monitor using the oscillometric method. Both the systolic BP (SBP) ≥ 140 mmHg and the diastolic BP (DBP) ≥ 90 mmHg was measured using a standard mercury sphygmomanometer. A 2-min interval was allowed before the blood pressure measurement to control for signs of clinical alert and “white-coat”-like phenomena.

##### 2.2.3.2 Smoking and physical activity

According to the CVD risk equations (Ferrario et al., 2005), smoking was defined as ≥1 cigarette per day and physical activity (PA) was defined as moderate to vigorous (≥150 min per week) according to the WHO (World Health Organization, 2010) recommendations.

##### 2.2.3.3 Obesity

Height (cm), weight (kg), waist circumference (cm), and blood pressure were measured by trained occupational health nurses. The waist circumference was measured in the horizontal plane of the iliac crest superior border keeping the subject in a standing position after a full expiration. BMI was calculated as weight (kg) divided by the square of height (m^2^).

##### 2.2.3.4 Metabolic syndrome

The metabolic syndrome (MetS) is broadly defined by a cluster of multiple metabolic abnormalities, including central obesity, hypertension, dyslipidemia, and insulin resistance. The criteria for MetS include 3+ of the following components: (1) elevated waist circumference (≥ 88 cm for women and ≥ 102 cm for men, (2) elevated TG (≥ 150 mg/dL) or being on treatment for dyslipidemia (statin and/or fibric acid derivative), (3) reduced HDL-C (< 40 mg/dL in men and < 50 mg/dL in women) or being on treatment for dyslipidemia (statin and/or fibric acid derivative), (4) elevated BP (systolic ≥ 130 and/or diastolic ≥ 85 mmHg) or antihypertensive drug treatment in patients with a history of hypertension), and (5) elevated FG (≥ 100 mg/dL) or drug treatment for elevated glucose level (Grundy et al., 2004).

#### 2.2.4 Biological markers

Standard techniques were used for assessing fasting blood glucose (FBG), total cholesterol (TC), high-density lipoprotein cholesterol (HDL-C) and triglycerides (TG). Fasting plasma homocysteine (Hcy) was measured by HemosIL™ Homocysteine assay, an automated, latex-enhanced immunoassay used for the quantitative determination of plasma Hcy (Instrumental Laboratory, Bedford, MA, USA). Lipoprotein(a) [Lp(a)] plasma concentrations were determined by IMMAGE® LPA immunonephelometric assay (IMMAGE LPA), performed on the IMMAGE® Immunochemistry System (Beckman Instrument, Inc., Galway, Ireland).

#### 2.2.5 Ten-year cardiovascular risk estimate

The 10-year CV risk was assessed using the CV risk score (CRS) developed within the Italian project CUORE (Epidemiology and Prevention of Ischemic Heart Diseases) (Ferrario et al., 2005; Palmieri et al., 2006). The CUORE project, coordinated by the Italian National Institute of Health, is a prospective fixed-cohort study that includes representative cohorts from all the Italian regions. The CUORE project risk scores assess the likelihood of experiencing a first CV event over the following 10 years by evaluating the level of eight risk factors: age, gender, SBP, TC, HDL-C, diabetes mellitus, smoking habit, and use of antihypertensive medication. The method was validated in patients aged 35 to 69 years without previous major CV accidents. According to CRS, the subjects are classified into low-risk (CRS < 3.0%), moderate-risk (CRS = 3.0–19.9%), or high-risk (CRS ≥ 20.0%) groups. Since high CV risk is uncommon in blood donors, our population was divided into two groups: low-risk (<3%) and moderate-to-high risk (≥3%). This threshold identifies individuals for whom annual monitoring of CV risk factors is recommended.^1^ Data were collected using the software CUORE.exe, free downloadable from the CUORE project website.^2^

The CRS showed similar discrimination power compared with Framingham and European SCORE equations but is more appropriate for the Italian population to avoid risk overestimation in a population with a low incidence of cardiovascular disease (Donfrancesco et al., 2010). The Italian Ministry of Health recommended the CRS score for cardiovascular risk assessment of the general adult Italian population in primary prevention (Donfrancesco et al., 2010; Giampaoli et al., 2007).

### 2.3 Statistical analysis

Between-group differences were evaluated with Pearson’s chi-square and Student’s t-tests. Partial eta-square (η^2^) and Cohen’s d values were computed to assess the effect size (Cohen, 1988). Cohen’s *d* = 0–0.2 is considered trivial, 0.2–0.5 small, 0.5–0.8 moderate, and ≥ 0.8 large. Conversely, *η*^2^ = 0.01–0.05 is considered small, 0.06–0.14 moderate, and > 0.14 large. Univariate logistic regression analyses were performed to assess the risk of moderate CRS. The risk was synthesized as crude odds ratios (ORs) with 95% confidence intervals (95% CI). Binary logistic regression models were implemented to estimate the role of alexithymia in predicting the level of CRS, adjusting for clinical, lifestyle, and sociodemographic variables. The variables included in the logistic regression model were selected for their well-established link with cardiometabolic diseases. The CV risk variables excluded from the regression models were those that were already included in the computation of our dependent variable (CRS: 10-year CV prediction algorithm). The CRS was considered the dependent variable (dummy coded: 0 = low CRS; 1 = moderate-to-high CRS). The sociodemographic (Step 1), lifestyle (Step 2), anthropometric and biological (Step 3), and psychological (Step 4) data were entered as independent variables in separate blocks to determine how well each variable predicted the outcome. Four regression steps were processed and regression coefficients, confidence intervals (CI), odds ratio (OR), and *p*-values were estimated. A two-sided *α* level of 0.05 was considered statistically significant. The statistical analysis was performed using the software Stata version 14.1 (Stata Corp., College Station, Texas, USA).

## 3 Results

### 3.1 Characteristics of the sample

Subjects were prevalently males (61.1%), aged 46.9 years (SD = 8.39), married (77.2%), middle-class (84.3%), with a high education level (14.2 years, SD = 3.60) (Table 1). As a confirmation of the expected overall good health status of the participants, most of the subjects were nonsmokers (81.3%), half of them reported regular physical activity (56.4%) and presented anthropometric, CVD risk factors, and biomarkers indices (BMI, waist circumference, FBG, BP, TC, HDL-C, TG, and Hcy) within the normal range. Consistently, TAS-20, BDI-II, and SF-12 scored within the normal range (Table 2).

**Table 1:** Socio-demographic characteristics of the study sample stratified for estimated 10-year cardiovascular disease risk (CRS).

Variable	Total sample (*N* = 1,161)	Low CRS (*N* = 911, 78.5%)	Moderate-to-high CRS (*N* = 250, 21.5%)	*x*^2^/t	*p*	d/ƞ^2^
Age	46.9 (8.39)	44.6 (7.41)	55.2 (6.11)	19.65	<0.001	1.57
Gender						
Men	710 (61.1%)	480 (52.7%)	230 (92%)	127.60	<0.001	0.85
Women	451 (39.9%)	431 (47.3%)	20 (8%)			
Marital status						
Unmarried	151 (13%)	134 (14.7%)	17 (6.8%)	12.13	0.002	0.10
Married	896 (77.2%)	694 (76.2%)	202 (80.8%)			
Divorced/separate	96 (9.8%)	83 (9.1%)	31 (12.4%)			
Education	14.2 (3.60)	14.4 (3.63)	13.5 (3.38)	3.28	0.001	0.25
SES (*n* = 1,021)						
Low	52 (5.1)	41 (5.1%)	11 (4.9%)	0.20	0.90	0.01
Middle	861 (84.3)	670 (84.1%)	191 (85.3%)			
High	108 (10.6)	86 (10.8%)	22 (9.8%)			

CRS, CUORE Risk Score; SES, Socio-Economic Status.

**Table 2:** Clinical characteristics of the study sample stratified for estimated 10-year cardiovascular disease risk (CRS).

Variable	Total sample (*N* = 1,161)	Low CRS (*N* = 911, 78.5%)	Moderate-to-high CRS (*N* = 250, 21.5%)	*x*^2^/t	*p*	d/ƞ^2^
*Lifestyle behavior*						
Smokers						
Yes	217 (18.7%)	143 (15.7%)	74 (29.6%)	24.95	<0.001	0.36
No	944 (81.3%)	768 (84.3%)	176 (70.4%)			
Cigarettes/day	2.2 (5.05)	1.4 (4.2)	3 (5.9)	4.90	<0.001	0.35
Regular physical activity						
Yes	655 (56.4%)	525 (57.6%)	130 (52%)	2.52	0.11	0.11
No	506 (43.6%)	386 (42.4%)	120 (48%)			
Physical activity/week	1.4 (1.3)	1.5 (1.7)	1.3 (1.5)	2.07	0.040	0.16
*Anthropometric and biological markers*						
BMI (Kg/m^2^)	25.9 (3.7)	25.6 (3.7)	26.3 (3.9)	2.44	0.014	0.16
Waist circumference (cm)	91.5 (11.2)	90.8 (11.9)	92.2 (11.9)	1.65	0.09	0.11
MetS	109 (9.4%)	40 (4.4%)	69 (27.6%)	117.25	<0.001	0.83
FPG (mg/dL)	98.1 (11.8)	97.5 (12.8)	98.6 (10.9)	1.50	0.13	0.10
SBP (mm/Hg)	120.8 (7.2)	120.5 (7.1)	120.8 (7.7)	0.70	0.47	0.05
DBP (mm/Hg)	75.8 (6.1)	75.4 (6.2)	76 (6.2)	1.46	0.14	0.10
TC (mg/dL)	199.9 (34.3)	195.8 (34.3)	203.7 (36.6)	3.41	<0.001	0.23
TG (mg/dL)	103.2 (68.1)	99.9 (63.4)	108.5 (86.9)	1.76	0.07	0.12
HDL-C (mg/dL)	54.4 (13)	54.5 (13)	54.8 (13)	0.04	0.96	0.01
LDL-C (mg/dL)	141.7 (35.6)	138.3 (36.3)	145.2 (36)	2.72	0.006	0.19
Hcy (μmol/L)	11.0 (4.8)	70 (7.9%)	34 (12.9%)	6.15	0.013	0.17
Lp(a) (mg/dL)	20.5 (24.4)	11 (4.4)	10.8 (4.5)	0.87	0.38	0.06
*Psychological data* (*n* = 1,021)						
TAS-20	41.4 (11.7)	40.7 (11.6)	43.8 (11.5)	3.50	<0.001	0.27
DIF	12.3 (5.8)	12.1 (5.7)	13.1 (5.8)	2.41	0.016	0.17
DDF	11.1 (4.5)	10.8 (4.5)	12 (4.1)	3.53	<0.001	0.27
EOT	17.9 (4.7)	17.8 (4.7)	18.6 (4.6)	2.21	0.027	0.17
BDI-II	6 (6.7)	5.9 (6.7)	6.2 (6.8)	0.44	0.66	0.04
SF-12						
PCS	51.9 (5.8)	52.4 (5.6)	52.2 (5.8)	0.47	0.64	0.04
MCS	48.5 (8.7)	48.5 (9.3)	48.9 (9.4)	0.56	0.57	0.04

BDI-II, Beck Depression Inventory-II; BMI, Body Mass Index; CRS, CUORE Risk Score; DDF, Difficulty Describing Feelings; DIF, Difficulty Identifying Feeling; DPB, Diastolic Blood Pressure; EOT, Externally-Oriented Thinking; FPG, Fasting Plasma Glucose; Hcy, Homocysteine; HDL-C, High Density Lipoprotein Cholesterol; LDL-C, Low Density Lipoprotein Cholesterol; Lp(a), Lipoprotein(a); MCS, Mental Component Summary; MetS, Metabolic syndrome; PCS, Physical Component Summary; SBP, Systolic Blood Pressure; SF-12, 12-item Short Form; TAS-20, 20-item Toronto Alexithymia Scale; TC, Total Cholesterol; TG, Triglycerides.

### 3.2 Comparison between subjects with moderate-to-high and low CRS

As expected, more than three quarters of the participants had CRS ≤ 3% (*n* = 911, 78.5%), whereas nobody was classified at high CVD risk (CRS ≥ 20.0%). Subjects with higher CRS were prevalently older (*d* = 1.57, *p* < 0.001), men (*d* = 0.85, *p* < 0.001), unmarried (*ƞ*^2^ = 0.10, *p* = 0.002), and had lower education (*d* = 0.25, *p* < 0.001) (Table 1). As we expected, most of them were smokers (*ƞ*^2^ = 0.36, *p* < 0.001) and had higher anthropometric and biological risk factors (BMI, *d* = 0.16, *p* = 0.014; MetS, *ƞ*^2^ = 0.83, *p* < 0.001; TC, *d* = 0.23, *p* < 0.001; LDL-C, *d* = 0.19, *p* = 0.006; Hcy, *d* = 0.17, *p* = 0.017). Overall, all the participants showed a low CV risk (women: mean CRS = 1.0%, prevalence = 95%; men: mean CRS = 2.85%, prevalence = 67.5%), which was about 3 times lower when compared with the Italian general population (mean CRS in women was 3.0% and men 8.3%) (Palmieri et al., 2011).

By comparing the psychological factors between higher and lower CVD risk subjects, only alexithymia resulted significantly higher in the higher CVD risk subjects, though having a small effect size (TAS-20 total, *d* = 0.27, *p* < 0.001; and factor scales: DIF, *d* = 0.17, *p* = 0.016; DDF, *d* = 0.27, *p* < 0.001; EOT, *d* = 0.17, *p* = 0.027) (Table 2).

### 3.3 Comparison between subjects with higher (HA) and lower (LA) alexithymia

As expected, compared with LA, HA subjects were significantly older (*d* = 0.20, *p* = 0.002), less educated (*d* = 0.42, *p* < 0.001). Moreover, HA subjects were more prevalent in the lower socio-economic level and less in the higher socio-economic level (*d* = 0.17, *p* < 0.001). Gender and marital status were evenly distributed in the two groups. In addition, compared with LA, HA subjects had expectedly more depressive symptoms (*d* = 1.03, *p* < 0.001) and showed greater psychosocial impairment by considering both the components of the SF-12 (MCS, *d* = 0.69, *p* < 0.001; PCS, *d* = 0.47, *p* < 0.001) (Tables 3, 4).

**Table 3:** Socio-demographic characteristics of the study population (*N* = 1,021), stratified for alexithymia.

Variable	Lower alexithymia (LA) (*N* = 785, 76.9%)	Higher alexithymia (HA) (*N* = 236, 23.1%)	*x*^2^/t	*p*	d/ƞ^2^
Age	45 (9.6)	47.1 (10)	3.07	0.002	0.20
Gender					
Men	287 (36.6%)	100 (42.4%)	2.60	0.11	0.05
Women	498 (63.4%)	136 (57.6%)			
Marital status^a^					
Unmarried	123 (15.7%)	36 (15.3%)	0.73	0.69	0.03
Married	585 (74.5%)	181 (76.7%)			
Divorced/separate	77 (9.8%)	19 (8.1%)			
Education	14.6 (3.6)	13.1 (3.3)	5.48	<0.001	0.42
SES (*n* = 1,021)					
Low	26 (3.3%)	26 (11%)	29.9	<0.001	0.17
Middle	663 (84.5%)	198 (83.9%)			
High	96 (12.2%)	12 (5.1%)			

SES, Socio-Economic Status.

**Table 4:** Clinical characteristics of the study population (*N* = 1,021), stratified for alexithymia.

Variable	Lower alexithymia (LA) (*N* = 785, 76.9%)	Higher alexithymia (HA) (*N* = 236, 23.1%)	*x*^2^/t	*p*	d/ƞ^2^
*Lifestyle behavior*					
Smokers					
Yes	146 (18.6%)	52 (22%)	1.37	0.26	0.03
No	639 (81.4%)	184 (78%)			
Cigarettes/day	1.8 (4.8)	2.2 (5.2)	1.29	0.19	0.08
Regular physical activity					
Yes	471 (60%)	112 (47.5%)	11.65	0.001	0.11
No	314 (40%)	124 (52.5%)			
Physical activity/week	1.6 (1.6)	1.3 (1.9)	1.98	0.047	0.12
*Anthropometric and biological markers*					
BMI (Kg/m^2^)	25.6 (3.7)	26.3 (3.9)	2.44	0.014	0.16
Waist circumference (cm)	90.8 (11.9)	92.2 (11.9)	1.65	0.09	0.11
MetS	67 (8.5%)	30 (12.7%)	3.68	0.058	0.06
FPG (mg/dL)	97.5 (12.8)	98.6 (10.9)	1.50	0.131	0.10
SBP (mm/Hg)	120.5 (7.1)	120.8 (7.7)	0.70	0.479	0.05
DBP (mm/Hg)	75.4 (6.2)	76 (6.2)	1.46	0.143	0.10
TC (mg/dL)	195.8 (34.3)	203.7 (36.6)	3.41	0.001	0.23
TG (mg/dL)	99.9 (63.4)	108.5 (86.9)	1.76	0.07	0.12
HDL-C (mg/dL)	54.5 (13)	54.8 (13)	0.04	0.96	0.01
LDL-C (mg/dL)	138.3 (36.3)	145.2 (36)	2.72	0.006	0.19
Hcy (μmol/L)	11 (4.4)	10.8 (4.5)	0.87	0.38	0.06
Lp(a) (mg/dL)	19.2 (22.8)	23.4 (28.1)	2.04	0.045	0.17
*Psychological data*					
BDI-II	4.4 (4.9)	10.7 (8.7)	15.20	<0.001	1.03
SF-12					
PCS	53.2 (5.1)	50.6 (6.7)	7.09	<0.001	0.47
MCS	50.1 (8.6)	43.8 (10.1)	10.08	<0.001	0.69

BDI-II, Beck Depression Inventory-II; BMI, Body Mass Index; CRS, CUORE Risk Score; DPB, Diastolic Blood Pressure; FPG, Fasting Plasma Glucose; Hcy, Homocysteine; HDL-C, High Density Lipoprotein Cholesterol; LDL-C, Low Density Lipoprotein Cholesterol; Lp(a), Lipoprotein(a); MCS, Mental Component Summary; MetS, Metabolic syndrome; PCS, Physical Component Summary; SBP, Systolic Blood Pressure; SES, Socioeconomic status; SF-12, 12-item Short Form; TC, Total Cholesterol; TG, Triglycerides.

Despite a small effect size, HA subjects had overall more unhealthy lifestyle behaviors (less physical activity and higher BMI, *d* = 0.11, *p* = 0.014, and *d* = 0.16, *p* < 0.001, respectively) and higher CVD risk factors such as TC (*d* = 0.23, *p* < 0.001), LDL-C (*d* = 0.19, *p* = 0.006), and Lp(a) (*d* = 0.17, *p* = 0.045) compared with LA subjects.

### 3.4 Predicting CVD risk from alexithymia

Table 5 shows binary logistic regression model with the 10-year CRS as a binary outcome criterion (low/high).

**Table 5:** Logistic regression model examining predictors of low and moderate-to-high 10-year cardiovascular disease risk (CRS) using the CUORE risk equation.

	Model 1 (*R*^2^ = 0.028)		Model 2 (*R*^2^ = 0.078)		Model 3 (*R*^2^ = 0.24)		Model 4 (*R*^2^ = 0.26)	
Predictor variable(s)	OR (95% CI)	*p*	OR (95% CI)	*p*	OR (95% CI)	*p*	OR (95% CI)	*p*
Marital status	1.50 (1.08–2.08)	0.016	1.58 (1.12–2.21)	0.008	1.63 (1.13–2.34)	0.009	1.67 (1.16–2.42)	0.006
Education	0.94 (0.89–0.98)	0.006	0.95 (0.90–0.99)	0.035	0.94 (0.89–0.99)	0.026	0.95 (0.90–1.01)	0.09
BMI			1.12 (1.08–1.18)	<0.001	1.05 (0.99–1.10)	0.23	1.05 (1–1.11)	0.06
Physical activity			1.05 (0.75–1.48)	0.005	1.01 (0.70–1.46)	0.94	1.03 (0.70–1.49)	0.89
MetS					1.15 (1.08–1.25)	<0.001	1.14 (1.08–1.25)	<0.001
LDL-C (mg/dL)					1.02 (1.01–1.02)	<0.001	1.02 (1.01–1.02)	<0.001
Hcy (μmol/L)					1.07 (1.03–1.11)	<0.001	1.07 (1.03–1.10)	0.001
SF-12 PCS							1.04 (1–1.08)	0.037
SF-12 MCS							1.01 (0.98–1.03)	0.50
BDI-II							1 (0.97–1.04)	0.78
TAS-20							1.02 (1.01–1.04)	0.009

BDI-II, Beck Depression Inventory-II; BMI, Body Mass Index; Hcy, Homocysteine; LDL-C, Low Density Lipoprotein Cholesterol; MetS, Metabolic syndrome; SF-12, 12-item Short Form; PCS, Physical Component Summary; MCS, Mental Component Summary; TAS-20, 20-item Toronto Alexithymia Scale.

Sociodemographic (marital status, education level), lifestyle (BMI, physical education), biological (MetS, LDL-C, Hcy) and psychological (quality of life, depressive symptoms, alexithymia) risk factors for CVD were used as independent variables.

In the first step (*R*^2^ = 0.03), being married and having a lower education were significantly associated with higher CVD risk (ORs = 1.50 and 0.94, respectively). In the second step (*R*^2^ = 0.08), marital status and education remained significant predictors (ORs = 1.58 and 0.95, respectively), while BMI and physical activity showed a significant positive association with high CVD risk (ORs = 1.12 and 1.05, respectively). In the third step (*R*^2^ = 0.24), marital status (OR = 1.63), education (OR = 0.94), BMI (OR = 1.05), and physical activity (OR = 1.01) remained key factors. Additionally, MetS emerged as a significant predictor of higher CVD risk (OR = 1.15), along with LDL-C (OR = 1.02) and Hcy (OR = 1.07). When the psychological variables were added in the fourth step (*R*^2^ = 0.26), the TAS-20 (OR = 1.02) significantly predicted higher CVD risk along with physical component of SF-12 (OR = 1.04), marital status (OR = 1.67), MetS (OR = 1.14), LDL-C (OR = 1.02), and Hcy (OR = 1.07).

In other words, for each point increase on the TAS-20, the odds of having a higher CVD risk increased by 2% (Table 5).

## 4 Discussion

The present study investigated whether alexithymia was associated with increased estimated CVD risk over 10 years in a healthy, low-risk blood donor population. Specifically, we compared sociodemographic, lifestyle, biological, and psychological factors between groups with different levels of CVD risk and alexithymia, and we investigated whether alexithymia contributes to CVD risk independently of established risk factors. As expected, participants with a higher CVD risk were older, more likely to be male, unmarried, and had lower education. They were also more likely to smoke, have a higher BMI, and show indicators like MetS, elevated LDL cholesterol, and higher homocysteine levels. Among the psychological factors, only alexithymia was notably higher in participants with greater CVD risk. Moreover, after accounting for other risk factors, alexithymia still independently predicted CVD risk. In summary, we found that even in a healthy, low-risk population, higher alexithymia was associated with an increased likelihood of higher CVD risk, beyond what could be explained by traditional CVD risk factors (like BMI, lifestyle, and biological markers).

Recently, several studies have notably changed the notion of the heart as a mechanic organ beating through an internal pacemaker and pumping blood into vessels and arteries. The heart is embedded in a complex network of organs and functions including the immune, endocrine, and nervous systems. The so called “heart-brain axis” indicates a feedback control network including afferent, efferent, and local well-known neural circuits (such as the hypothalamic–pituitary axis triggering a neuro-hormonal cascade toward the autonomic nervous system) as well as brain circuits (such as the insular cortex, the anterior cingulate cortex (ACC), and the amygdala), (for a review see Fioranelli et al., 2018; Jha et al., 2019). Not surprisingly, therefore, mental disorders (particularly depression) and psychosocial stress have been repeatedly found as strictly linked in a bidirectional way to CVD onset, maintenance, and prognosis (Gaffey et al., 2022; O’Riordan et al., 2023). Studies have reported a connection through mediational factors such as social (unemployment, substance abuse, social isolation, and low education), biological (autonomic dysregulation, platelet factors, endothelial function, circulating neurohormones, insulin resistance, inflammatory cytokines), and behavioral mechanisms (obesity, smoking, low physical activity, poor diet, and medical noncompliance) both in large prospective surveys (Song et al., 2019) and in systematic reviews (Albasheer et al., 2024; Bradley and Rumsfeld, 2015; Gaffey et al., 2022). In addition, stimulation studies have documented the effects of cingulate electrical stimulation on autonomic and endocrine functions (Alexander et al., 2019; Koelsch et al., 2013) triggering altered emotional and behavioral responses such as the “cardiac signature of emotionality” (Koelsch et al., 2007).

The main strength of this study is the use of a large specific sample of individuals showing above average somatic and psychosocial health, as documented by the biological and psychological assessments. Thus, the subjects involved showed a baseline low-risk profile for CVD. The recruited subjects were middle-class, highly educated, with low exposure to known lifestyle (smoking, low physical activity) and biomedical (blood pressure, BMI, glycemia, cholesterol, triglycerides, homocysteine, lipoprotein(a), and metabolic syndrome) risk factors. Moreover, they had normal levels of alexithymia, depression, and psychosocial functioning. Nonetheless, HA subjects still presented a significantly higher risk for moderate CVD, as compared with the estimated 10-year CV risk score. More specifically, for every 1-point increase in the TAS-20 scores there was a significant and independent 2% increased risk of CVD over an estimated follow-up period of 10-years, even after adjusting for socio-demographic, established biomedical, and relevant psychological factors. In the fully adjusted model, although significant, only a small portion of variance was explained by the predicting variables. However, it is noteworthy that a personality construct (alexithymia) not directly associated with the biomedical outcome (CRS) showed a similar OR as the traditional biomedical and lifestyle risk factors (smoking, LDL, Hcy) or even higher (BMI, physical activity, depression) (e.g., O’Riordan et al., 2023). Furthermore, this result seems more surprising if one considers that the investigated sample was at a very low risk profile level from either the physiological, lifestyle, and psychological viewpoints.

Even though less investigated than other psychological constructs such as depression, alexithymia is proved to be involved in many mediational mechanisms underlying the health status. A poor cognitive processing of feelings affects negatively the regulation of the biological systems in the body (Luminet et al., 2018; Porcelli and Taylor, 2018). Alexithymia was found as a potential CV risk indicator in several studies, suggesting a connection with CV-related conditions as hypertension, atherosclerosis, CVD, and CV mortality both in general and clinical populations (e.g., Carta et al., 2022; Tolmunen et al., 2010; Vadini et al., 2019). In addition, several CV risk factors, such as sympathetic over-reactivity (Bermond et al., 2010), inflammation (Honkalampi et al., 2011), diabetes (Fares et al., 2019; Luca et al., 2015), obesity/MetS (Conti et al., 2019; Conti et al., 2021; Conti et al., 2023), and atherosclerosis (Grabe et al., 2010; Vadini et al., 2019) have been associated with a higher prevalence of alexithymia. In a series of experimental studies on healthy subjects, Koelsch et al. (2007, 2013) combined the use of electrocardiogram (ECG) with functional magnetic resonance imaging (fMRI) during the processing of emotional stimuli. The studies found a “cardiac signature of emotionality” involving a specific amplitude in the resting ECG correlating with brain activity in the amygdala and the hippocampus, with alexithymia scores, and with heart rate variability (HRV). Systematic reviews have shown that alexithymia may predict hypertension and subclinical atherosclerosis, may be a risk factor for early death in the long-term course of post-myocardial infarction, as well as may predispose to unhealthy behaviors including dysfunctional health care-seeking behavior in the event of adverse cardiac events (Montisci et al., 2023; Sancassiani et al., 2023).

Potential mechanisms underlying the relationship between alexithymia and CV risk are far from being definite. The CV network includes other biological systems such as the immune and the nervous (Fioranelli et al., 2018; Jha et al., 2019), thus there is some evidence that alexithymia may affect both systems. To this extent, some studies have shown that alexithymia is associated with increased inflammation and altered profiles of circulating cytokines, although no clear shift toward pro- or anti-inflammatory mediators has been demonstrated (Kano et al., 2018). In the Kuopio Depression Study, Honkalampi and colleagues (Honkalampi et al., 2011) found that alexithymia was associated with high-sensitive C-reactive protein (hsCRP) (a protein generated by the liver in response to IL-6 secretion by macrophages and T cells) as well as with adiponectin (a cytokine expressed in the adipose tissue). Individuals with major depression and CVD show a similar association pattern. Consistent with our findings, De Berardis et al. (2008) found significant connections between alexithymia, higher hsCRP, and altered serum lipid levels (TC, HDL, and LDL) in a group of drug-naïve major depressed outpatients. Altered neural activity in the limbic brain areas has been found in HA subjects and may constitute another potential mechanism linking cognitive processing and subjective experience of emotions to CV activity. Neuroimaging studies found out that altered functional activity of amygdala, insula, and ACC (Goerlich and Aleman, 2018; Moriguchi and Komaki, 2013) together with a reduced volume of dorsal ACC, left amygdala, and insula (Xu et al., 2018) may be considered the neural basis of alexithymia. Alexithymia may mirror a cluster of psychosocial, behavioral and biological risk factors that have synergistic effects on the individual health. Consistent with this hypothesis, even if presenting with a low-risk profile, our cohort of blood donors with HA reported relatively higher indices of unhealthy behaviors such as sedentary lifestyle, MetS, BMI, TC, HD, circulating Lp(a), as well as older age, lower education level, higher unemployment rate, lower socio-economic status, higher levels of depressive symptoms and overall psychosocial functioning. All those factors are considered alexithymia-related risk factors mediating between general health and CV mortality (Montisci et al., 2023; Sancassiani et al., 2023).

The results of our study have some limitations. First and foremost, the cross-sectional design does not allow addressing causality. Our data may indicate that alexithymia contributes to CVD risk, but a number of risk factors (low education, poor socio-economic status, depressive symptoms, and lower psychosocial functioning) may affect the individual ability to manage self-regulation. Longitudinal studies are needed to further assess those associations. Second, alexithymia may also be partly secondary to depression (Sagar et al., 2021). In our sample, a past or current diagnosis of major depression was not screened out at the enrollment stage, though alexithymia was then significantly associated with depressive symptoms and a poorer mental component of psychosocial functioning. Therefore, we cannot rule out that depression may play a relevant role in the association with CVD risk, even though adjusting for depressive symptoms did not decrease CRS risk score. Third, some variables that may mediate the relationship between alexithymia and CVD risk, such as psychopathology and social isolation, were not assessed. It has been suggested that alexithymia may affect CVD through social and emotional pathways rather than directly through behavioral or physiological factors (Montisci et al., 2023; Sancassiani et al., 2023). Our findings partly support these observations, although blood donors typically show healthy lifestyle and prosocial motivation (Li et al., 2015). In addition, adjusting for lifestyle factors did not change the predicting role of alexithymia. Finally, alexithymia was assessed using only a self-report scale, even though a multimethod assessment including facets of the constructs from other sources of data (as clinician ratings, by-proxy information, and implicit motives) could be preferred (Sekely et al., 2018). However, it is widely recognized that TAS-20 captures the core dimensions of the construct (impairment in experiencing and describing feelings) (Bagby et al., 2020) and shows overtime absolute and relative stability in the general population (Hiirola et al., 2017). Therefore, the self-report scale used in the study can be considered a valid and sound measure of alexithymia.

Despite these limitations, the present findings indicate that alexithymia is an independent strong predictor of CVD risk even in above average healthy individuals. Further research is needed to confirm longitudinally this association and to examine the underlying mechanisms. In addition, while previous studies have mainly focused on high-risk or clinical populations, our results showed that alexithymia could be an early indicator of cardiovascular disease risk, adding a psychological dimension to cardiovascular prevention efforts. Since alexithymia seems linked to cardiovascular disease risk even in low-risk samples, psychological factors such as emotion regulation could be important additions to traditional cardiovascular disease risk screenings. Further longitudinal research is needed to confirm this association and to examine the underlying mechanisms.

## Data Availability

The data analyzed in this study is subject to the following licenses/restrictions: the raw data supporting the conclusions of this article will be made available by the authors, without undue reservation. Requests to access these datasets should be directed to roberta.lanzara@unich.it.

## Footnotes

^1^ www.cuore.iss.it

^2^ http://www.cuore.iss.it/eng/
